# Evolocumab lowers LDL-C safely and effectively when self-administered in the at-home setting

**DOI:** 10.1186/s40064-016-1892-3

**Published:** 2016-03-09

**Authors:** Ricardo Dent, Raju Joshi, C. Stephen Djedjos, Jason Legg, Mary Elliott, Michelle Geller, Dawn Meyer, Ransi Somaratne, Chris Recknor, Robert Weiss

**Affiliations:** Amgen (Europe) GmbH, Dammstrasse 23, 6300 Zug, Switzerland; Amgen Inc., One Amgen Center Dr, Thousand Oaks, CA 91320 USA; Amgen Ltd., 240 Cambridge Science Park, Milton, Cambridge, CB4 0WD UK; Centers for Advanced Research and Education, 2350 Limestone Pkwy NE, Gainesville, GA 30501 USA; Maine Research Associates, 2 Great Falls Plaza, Auburn, ME 04210 USA

**Keywords:** Evolocumab, PCSK9 inhibition, Home-use, In-clinic, Self-administration

## Abstract

Evolocumab has been shown to consistently reduce low-density lipoprotein cholesterol (LDL-C) across populations. The phase 3 studies included administration in the home-use and in-clinic settings but did not specifically evaluate the feasibility of home-use administration. Two clinical studies enrolled patients with hypercholesterolemia or mixed dyslipidemia on statin therapy and with/without ezetimibe received evolocumab in the home-use setting. Patients were randomized to self-administer evolocumab using one of two injection devices biweekly over 6 weeks (autoinjector or prefilled syringe; n = 149; ClinicalTrials.gov, NCT01849497) or monthly over 12 weeks (autoinjector or automated minidoser; n = 164; NCT01879319). The first self-administration occurred in the in-clinic setting, and two more were performed in the at-home setting. Patients were successful in self-administering evolocumab in the home-use setting in approximately 95 % of attempts and experienced LDL-C reductions from baseline to week 6 or the mean of weeks 10 and 12 of approximately 65 %. Rates of successful self-administration and LDL-C reduction were similar across dosing schedules and study devices. Adverse events were similar between randomized groups and generally mild in severity. In two clinical studies, therefore, patients were able to successfully self-administer evolocumab in both the in-clinic and at-home settings regardless of which dosing schedule or device they used.

**Electronic supplementary material:**

The online version of this article (doi:10.1186/s40064-016-1892-3) contains supplementary material, which is available to authorized users.

## Background

Evolocumab (AMG 145; Repatha^®^, Amgen Inc, Thousand Oaks, CA) is a monoclonal antibody administered via subcutaneous injection that inhibits proprotein convertase subtilisin/kexin type 9 (PCSK9) and has been shown to decrease low-density lipoprotein cholesterol (LDL-C) levels significantly and consistently across multiple populations with hypercholesterolemia (Koren et al. 2014; Raal et al. 2015; Robinson et al. 2014; Sabatine et al. 2015; Stroes et al. 2014; Blom et al. 2014). Evolocumab is approved for at-home administration by patients or their caregivers either 140 mg biweekly (administered via a 1 mL autoinjector) or 420 mg monthly (three 1 mL autoinjectors or one 3.5 mL automated minidoser [AMD]) (Amgen Inc 2015c). These two different dosing regimens provide similar LDL-C reduction, are clinically equivalent (Koren et al. 2014; Raal et al. 2015; Robinson et al. 2014; Sabatine et al. 2015; Stroes et al. 2014), and are offered to accommodate patient preference.

Monoclonal antibodies self-administered in the home-use setting are relatively new for the treatment of hypercholesterolemia (Rader and Kastelein 2014). The LDL-C reduction and safety profile observed with PCSK9 inhibition [55–75 % in the evolocumab clinical program (Koren et al. 2014; Raal et al. 2015; Robinson et al. 2014; Stroes et al. 2014; Sabatine et al. 2015; Blom et al. 2014)] presents compelling reason for eligible patients to initiate home-use administration of an injectable medication. Moreover, subcutaneously-injected biologics are widely used by patients with diseases such as rheumatoid arthritis, psoriasis, and osteoporosis in the home-use setting (Foltz et al. 2013; Hanley et al. 2012; American Academy of Dermatology Work Group et al. 2011).

Amgen’s evolocumab phase 2 and 3 development program, known as Program to Reduce LDL-C and cardiovascular Outcomes Following Inhibition of PCSK9 In different pOpulations (PROFICIO), reflects LDL-C reduction via PCSK9 inhibition in both the clinic and at-home settings. This program is composed of several completed studies that demonstrated safety and efficacy in hyperlipidemia and mixed dyslipidemia patients, as well as several ongoing studies that are evaluating safety and efficacy in the setting of atherosclerosis and secondary prevention (ClinicalTrials.gov, NCT01764633, NCT01813422, and NCT01984424). In the PROFICIO phase 2 studies (Stein et al. 2014), evolocumab was administered in the clinic by the healthcare practitioner, while in the 12-week, phase 3 studies (Koren et al. 2014; Raal et al. 2015; Robinson et al. 2014; Stroes et al. 2014), the initial one or two doses and the last dose were administered in the clinic (either by site, patient, or caregiver) and all other injections were administered at home by the patient or caregiver. Study drug and device safety were assessed separately via reports of AEs and adverse device effects, respectively. Though the program design included both healthcare professional–supervised administration and home-use administration, these studies did not specifically examine whether patients could successfully self-administer evolocumab in the home-use setting.

In addition to the aforementioned program-wide assessment of home vs in-clinic administration, we performed two randomized studies (THOMAS-1 and THOMAS-2) with similar designs that specifically evaluated injection success of evolocumab in the home-use setting with three different devices. In this article, we report the primary results of the THOMAS studies.

## Methods

THOMAS-1 (NCT01849497) and THOMAS-2 (NCT01879319) were multicenter, open label, parallel-arm, randomized studies that enrolled patients at 22 and 23 sites (respectively) in the United States and Canada from April to December 2013. Patients with hypercholesterolemia or mixed dyslipidemia on statin therapy with or without ezetimibe were eligible. Enrolled patients were randomized to receive evolocumab administered at home with the prefilled SureClick^®^ autoinjector vs a prefilled syringe (PFS) (THOMAS-1) or the prefilled SureClick^®^ autoinjector vs an AMD (THOMAS-2).

### Ethics, consent, and permissions

Both studies required written informed consent, adherence to Good Clinical Practices and local regulations, and approval of the protocol by institutional review boards/ethics committees.

### Patient enrollment and study procedures

Patients were eligible for enrollment in the THOMAS studies if they were 18–80 years of age, taking an approved statin (with or without ezetimibe) at a stable dose for at least 4 weeks before LDL-C screening, and had a fasting LDL-C ≥85 mg/dL and fasting triglycerides ≤400 mg/dL. Exclusion criteria included the following conditions or procedures: family or personal history of hereditary muscular disorders, moderate to severe heart failure, moderate to severe renal dysfunction, recent cardiovascular event or deep vein thrombosis/pulmonary embolism, recent or planned revascularization/cardiac surgery, type 1 diabetes, uncontrolled or recently-diagnosed type 2 diabetes, infection, recent malignancy, and major cardiac, endocrine, major hematologic, renal, metabolic, and gastrointestinal disorders. Patients were excluded for current or recent use of the following drugs: lipid-lowering drugs or supplements other than statins or ezetimibe, drugs or supplements that potentially alter lipid metabolism or inhibit cytochrome p450 family 3 subfamily A (CYP3A), and evolocumab or other PCSK9 inhibitor. Female patients were excluded if they were pregnant, breast-feeding, intended to become pregnant, or were of childbearing potential and not using effective birth control. Finally, patients were excluded if they were enrolled in another investigational device or drug study.

During screening prospective enrollees received 1 (THOMAS-1) or 3 (THOMAS-2) 1 mL placebo injections delivered via the PFS (THOMAS-1) or autoinjector (THOMAS-2) to confirm tolerance of subcutaneous injection; this period also included laboratory testing to confirm study eligibility. Patients who tolerated the screening and met eligibility criteria were randomized. Enrolled patients were randomized 1:1 by interactive voice/web-based response system to the autoinjector or PFS in THOMAS-1 and autoinjector or AMD in THOMAS-2. Randomization was stratified by baseline LDL-C <130 mg/dL vs ≥130 mg/dL.

Three total doses were administered over 6 weeks in THOMAS-1 (140 mg biweekly) and 12 weeks in THOMAS-2 (420 mg monthly). At the time of the first dose of evolocumab, the clinic healthcare practitioner trained the patient on how to use the device to safely deliver a full dose and reviewed the device’s instructions for use (see Additional file 1: Appendix for full training checklist). If applicable, the patient’s designee or caregiver was trained by the clinic staff on how to administer evolocumab with the study device. AEs, including adverse device effects, and device complaints were collected at all visits.

The first evolocumab dose was administered in the clinic under supervision of the staff, and the remaining doses were self-administered at home with the home-use device(s) the patient was randomized to. At the first visit (day 1) and during telephone contacts (weeks 2 and 4 in THOMAS-1, 4 and 8 in THOMAS-2), clinic staff documented whether the patient was able to administer a full dose of evolocumab. Patients returned to the site for LDL-C measurement at week 6 in THOMAS-1 and weeks 10 and 12 in THOMAS-2. The end of study visit was a telephone contact at week 8 for documenting AEs in THOMAS-1 and an in-clinic visit at week 12 in THOMAS-2.

### Study drugs and devices

Evolocumab is a sterile, preservative-free solution. Investigators could prescribe any concomitant medications or treatments except for prohibited drugs or supplements that potentially lower lipids, affect lipid metabolism, or inhibit CYP3A.

The autoinjector used in the phase 3 evolocumab studies contained 140 mg of evolocumab in 1 mL total solution. The PFS, similarly, contained 140 mg of evolocumab. Each autoinjector or PFS injection was administered in up to three anatomical locations: abdomen, thigh, or outer arm (arm injections required caregiver assistance).

The AMD is a single-use, disposable, on-body electromechanical injection device. It was packaged with a prefilled cartridge assembly containing 420 mg of evolocumab in a 3.5 mL solution. All AMD injections were administered to the patient’s abdomen, thigh, or outer arm with arm injections requiring caregiver assistance.

Diagrams of the devices are displayed in the Appendix of Additional file 1.

### Assessments

Successful administration of a full evolocumab dose in the at-home setting was assessed in telephone contacts at weeks 2 and 4 of THOMAS-1 and 4 and 8 of THOMAS-2.

Fasting lipid assessments were taken at screening and day 1 in both THOMAS studies, week 6 in THOMAS-1, and weeks 10 and week 12 in THOMAS-2. LDL-C was determined in a reflexive manner by using the Friedewald equation (Friedewald et al. 1972) unless the calculated LDL-C was <40 mg/dL or triglycerides were >400 mg/dL, in which case ultracentrifugation was used.

Blood samples were collected at day 1 and week 6 in THOMAS-1 and weeks 10 and 12 in THOMAS-2 to evaluate evolocumab concentration levels (pharmacokinetics [PK]) and PCSK9 levels (pharmacodynamics [PD]), as well as anti-evolocumab antibodies. PK and PD analyses were conducted and presented descriptively by study device. If samples tested positive for binding anti-evolocumab antibodies, they were also tested for neutralizing antibodies.

AEs and serious AEs were documented at all visits. Treatment-emergent AEs were defined as those occurring with an onset after the first administration of study drug and before the end of study visit, and investigators were asked to assess whether there was a reasonable possibility that the events were related to evolocumab or device. Adverse device effects were treatment-emergent AEs that the investigator deemed possibly related to the device. Investigators were trained about intentional use before enrolling patients.

Deaths and cardiovascular events were adjudicated by an independent clinical endpoint committee (TIMI Study Group, Boston, MA) composed of independent members blinded to patient identity, treatment assignment, and LDL-C concentrations.

### Endpoints

The primary endpoint of both THOMAS studies was the patient-reported successful outcome of attempted self-administered full-dose of evolocumab in the home-use setting with the study device (autoinjector or PFS in THOMAS-1, autoinjector or AMD in THOMAS-2). The secondary endpoint was the mean change in LDL-C from baseline to 6 weeks in THOMAS-1 and the mean of weeks 10 and 12 in THOMAS-2.

### Statistical analysis

The THOMAS studies did not test a formal hypothesis. For the analysis of the primary endpoint in both studies, the proportion of patients who successfully self-administered a full dose of evolocumab at each of the two home visits was reported. The 95 % confidence intervals (CIs) for the proportions were calculated using the Wilson score interval. The secondary endpoint was estimated percent change from baseline in LDL-C to week 6 in THOMAS-1 and the mean of weeks 10 and 12 in THOMAS-2. In THOMAS-1, this outcome was estimated using an analysis of covariance model that included study device and LDL-C stratum (i.e., <130 mg/dL vs ≥130 mg/dL). In THOMAS-2, the outcome was estimated using a repeated-measures mixed-effects model that included the screening LDL-C stratum, study device, visit, and study device by visit. Prespecified subgroup analyses included age (<65 years and ≥65 years) and sex. AEs, deaths, and adjudicated cardiovascular events were summarized and are presented descriptively. Analyses were conducted using SAS version 9.2 or later (SAS Institute, Cary, NC).

## Results

In the THOMAS-1 study, 271 patients were screened, 149 were randomized (autoinjector: n = 74, PFS: n = 75), and 144 completed the study (97 %). In the THOMAS-2 study, 243 patients were screened, 164 were randomized (autoinjector: n = 82, AMD: n = 82), and 157 completed the study (96 %). Patient disposition is displayed in the Appendix of Additional file 1.

The patient profiles of THOMAS-1 and THOMAS-2 were similar and balanced between study device groups (Table 1): 45 % of patients from both studies were women, the mean age was 60 years, 19 % had coronary artery disease, 22 % had type 2 diabetes mellitus, 43 % were National Cholesterol Education Program–high risk, and 11 % had cerebrovascular or peripheral artery disease.

**Table 1:** Baseline demographics and disease history of patients

	THOMAS-1		THOMAS-2	
	Autoinjector (n = 74)	PFS (n = 75)	Autoinjector (n = 82)	AMD (n = 82)
Mean (SD) age, years	60.6 (9.6)	61.2 (11.1)	59.2 (10.0)	60.1 (10.5)
Age group				
<65 years	46 (62)	42 (56)	57 (70)	49 (60)
≥65 years	28 (38)	33 (44)	25 (30)	33 (40)
Female sex	26 (35)	36 (48)	39 (48)	39 (48)
Race				
White	58 (78)	62 (83)	75 (92)	69 (84)
Black or African American	7 (10)	11 (15)	2 (2)	6 (7)
Asian	7 (10)	2 (3)	3 (4)	4 (5)
Other	1 (1)	0	2 (2)	3 (4)
Missing	1 (1)	0	0	0
Hispanic ethnicity	6 (8)	9 (12)	6 (7)	4 (5)
Baseline ezetimibe use	8 (11)	1 (1)	6 (7)	8 (10)
NCEP high risk	38 (51)	31 (41)	29 (35)	37 (45)
NCEP moderate/moderately high risk	25 (34)	29 (39)	36 (44)	33 (40)
Coronary artery disease	20 (27)	13 (17)	13 (16)	12 (15)
Type 2 diabetes mellitus	18 (24)	17 (23)	18 (22)	17 (21)
Hypertension	54 (73)	49 (65)	49 (60)	56 (68)
Cerebrovascular or peripheral artery disease	6 (8)	8 (11)	6 (7)	14 (17)
Mean (SD) baseline LDL-C, mg/dL^a^	118.1 (28.7)	116.9 (25.0)	117.3 (23.9)	115.3 (27.0)
Mean (SD) HDL-C, mg/dL	50.6 (13.3)	52.1 (14.7)	49.7 (14.6)	50.9 (14.5)
Mean (SD) triglycerides, mg/dL	143.5 (66.2)	138.1 (60.4)	139.9 (48.4)	147.5 (64.8)
Mean (SD) non-HDL-C, mg/dL	146.8 (33.2)	144.6 (30.2)	145.3 (26.3)	144.8 (30.9)

Values are presented as number (percent) of patients unless otherwise noted. Percentages may not add up to 100 owing to rounding

*AMD* automated minidoser, *HDL-C* high-density lipoprotein cholesterol, *LDL-C* low-density lipoprotein cholesterol, *NCEP* National Cholesterol Education Program, *non-HDL-C* non–high density lipoprotein cholesterol, *PFS* prefilled syringe, *SD* standard deviation

^a^LDL-C was based on calculated values unless calculated LDL-C was <40 mg/dL or triglycerides were >400 mg/dL, in which case the ultracentrifugation LDL-C value from the same blood sample was used instead, if available

### Efficacy

**Fig. 1:**
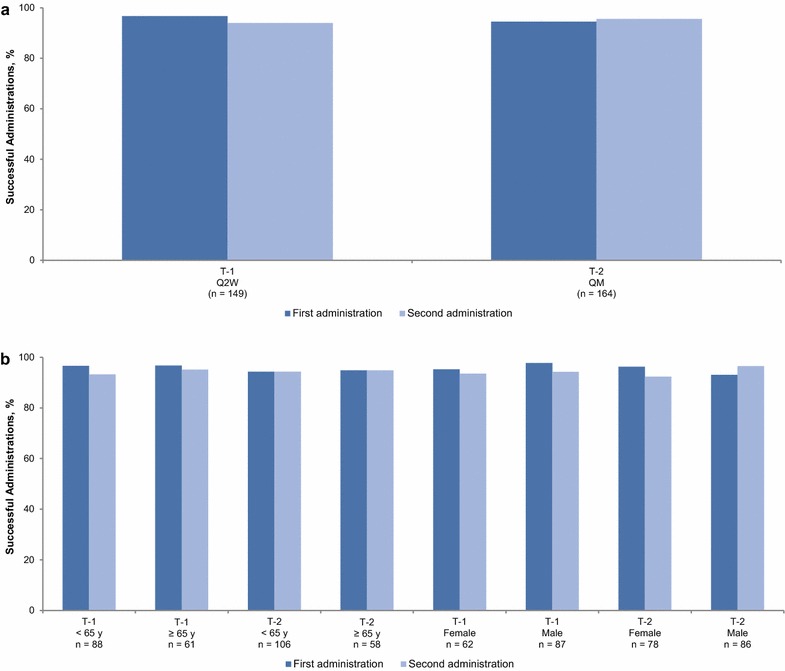
Proportion of successful home administrations in **a** overall population and **b** prespecified subgroups. *Q2W* biweekly (140 mg), *QM* monthly (420 mg), *T-1* THOMAS-1, *T-2* THOMAS-2

Patients were able to successfully self-administer a full dose of evolocumab in approximately 95 % of total attempts (Fig. 1a) in both studies. Rates of successful self-administration were similar regardless of the patients’ dosing schedule (Fig. 1a) or study device (Table 2). Subgroup analysis of the primary endpoint by screening age and sex (Fig. 1b) did not indicate any difference between subgroups in the ability to use either device.

**Table 2:** Successful home administration of evolocumab in THOMAS-1 and THOMAS-2

	THOMAS-1		THOMAS-2	
	Autoinjector (n = 74)	PFS (n = 75)	Autoinjector (n = 82)	AMD (n = 82)
First administration^a^				
No. successful administrations	71	73	77	78
% (95 % CI)	95.9 (88.7, 98.6)	97.3 (90.8, 99.3)	93.9 (86.5, 97.4)	95.1 (88.1, 98.1)
Second administration^b^				
No. successful administrations	68	72	76	79
% (95 % CI)	91.9 (83.4, 96.2)	96.0 (88.9, 98.6)	92.7 (84.9, 96.6)	96.3 (89.8, 98.7)

*AMD* automated minidoser, *CI* confidence interval, *PFS* prefilled syringe

^a^Week 2 in THOMAS-1, week 4 in THOMAS-2

^b^Week 4 in THOMAS-1, week 8 in THOMAS-2

**Fig. 2:**
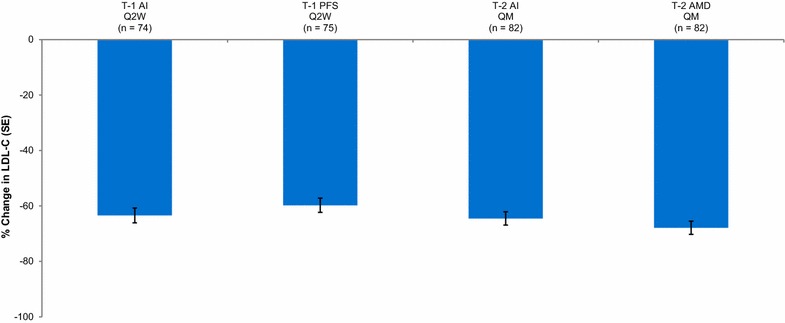
Change in LDL-C from baseline to 6 weeks in THOMAS-1 and to the mean of weeks 10 and 12 in THOMAS-2. LDL-C was based on calculated values unless calculated LDL-C was <40 mg/dL or triglycerides were >400 mg/dL, in which case the ultracentrifugation LDL-C value from the same blood sample was used instead, if available. *AI* autoinjector, *AMD* automated minidoser, *LDL-C* low-density lipoprotein cholesterol, *PFS* prefilled syringe, *Q2W* biweekly (140 mg), *QM* monthly (420 mg), *SE* standard error, *T-1* THOMAS-1, *T-2* THOMAS-2

LDL-C was reduced from baseline to week 6 (THOMAS-1) or the mean of weeks 10 and 12 (THOMAS-2) similarly regardless of the patients’ device or dosing schedule (Fig. 2). The estimated least squares mean change (95 % CI) in THOMAS-1 was −63.4 % (−68.7, −58.2) for the autoinjector group and −59.7 % (−64.8, −54.7) for the PFS group. In THOMAS-2, the estimated least squares mean change (95 % CI) was −64.5 % (−69.2, −59.8) for the autoinjector group and −67.9 % (−72.6, −63.2) for the AMD group.

Unbound evolocumab serum (PK) and PCSK9 levels (PD) were similar between devices in the THOMAS studies (Additional file 1: Supplementary Table 1).

### Safety

Safety data from the THOMAS studies is displayed in Table 3. Overall, the AEs in the THOMAS studies were of low frequency and severity and were similar between groups (25.6–32.9 % for AEs, 0–1.4 % for serious AEs). The most commonly occurring AEs in THOMAS-1 were headache (4.0 %, n = 6), bronchitis (2.0 %, n = 3), and abdominal pain (2.0 %, n = 3). The most commonly occurring AEs in THOMAS-2 were pain in extremity, fatigue, and sinusitis (all 2.0 %, n = 3).

**Table 3:** Safety in THOMAS-1 and THOMAS-2

	THOMAS-1		THOMAS-2		All
	Autoinjector (n = 74)	PFS (n = 75)	Autoinjector (n = 82)	AMD (n = 82)	(n = 313)
Total treatment-emergent AEs	20 (27.0)	22 (29.3)	27 (32.9)	21 (25.6)	90 (28.8)
Grade 3 or 4	2 (2.7)	3 (4.0)	1 (1.2)	1 (1.2)	7 (2.2)
AEs leading to study drug discontinuation	1 (1.4)	2 (2.7)	1 (1.2)	0	4 (1.3)
Serious	1 (1.4)	1 (1.3)	0	0	2 (0.7)
Nonserious	0	1 (1.3)	1 (1.2)	0	2 (0.7)
Serious AEs	2 (2.7)	3 (4.0)	1 (1.2)	0	6 (1.9)
Adverse device effects	0	0	2 (2.4)	1 (1.2)	3 (1.0)
Injection-site reactions	0	0	0	1 (1.2)	1 (0.3)

Values are presented as number (percent) of patients

*AEs* adverse events, *AMD* automated minidoser, *PFS* prefilled syringe

There were five total AEs in four patients leading to study drug discontinuation (two serious; n = 2 in autoinjector group, n = 1 in PFS group, THOMAS-1; n = 1 in autoinjector group, THOMAS-2) and no preferred term was reported more than once for these events. In THOMAS-1, one patient (autoinjector group) experienced cerebrovascular accident (serious AE), one subject (PFS group) experienced cholecystitis (serious AE) and cholelithiasis, and one subject (PFS group) experienced renal impairment. These events were not considered related to the study drug or device. In THOMAS-2, one patient (autoinjector group) experienced injection-site hematoma that was not considered related to the study drug but was considered related to the study procedures and device.

There were four adverse device effects total, all of which occurred in THOMAS-2 and were low severity (grade 1). One patient experienced two instances of injection-site reaction (AMD group); one patient, pain in extremity (autoinjector group); and one patient, injection-site hematoma (autoinjector group). There were no deaths in either study. There was one positively adjudicated cardiovascular event; one patient (THOMAS-1, autoinjector group) experienced an adjudicated, nonfatal ischemic stroke. No binding anti-evolocumab antibodies developed during evolocumab treatment.

## Discussion

The results of the THOMAS studies indicate patients can successfully administer evolocumab in the home-use setting without healthcare provider–supervision. The study devices were all comparable with respect to the primary endpoint of successful home-use self-administration as well as their safety profiles, with comparable rates of injection-site reactions and adverse device effects. Reduction in LDL-C following home-use administration was comparable across dosing regimens and devices. The devices tested were safe and well tolerated. AEs in the THOMAS studies were similar to AEs of the overall PROFICIO population (Koren et al. 2014; Raal et al. 2015; Robinson et al. 2014; Sabatine et al. 2015; Stroes et al. 2014; Blom et al. 2014; Stein et al. 2014). Very few adverse device effects were reported; also, these events were similar between groups and all grade 1 in severity.

Despite the availability of injectable biologics in the home-use setting for multiple other conditions (AbbVie Inc 2015; Amgen Inc 2015a, 2015b; Janssen Biotech Inc 2015; Novo Nordisk A/S 2015), injectable treatments are fairly new for treatment of dyslipidemia. In 2015, evolocumab and alirocumab were approved for treatment of hyperlipidemia and mixed dyslipidemia (Amgen Inc 2015c; Genzyme Corp 2015; Regeneron Pharmaceuticals Inc/sanofi-aventis US LLC 2015). Evolocumab is currently approved for both monthly and biweekly dosing (Amgen Inc 2015c), and alirocumab is approved for biweekly dosing (Regeneron Pharmaceuticals Inc/sanofi-aventis US LLC 2015). The addition of a monthly dosing option is intended to accomodate patient convenience. Evolocumab biweekly dosing involves an autoinjector (AI) or PFS that administers 1 mL. Most of the phase 3 evolocumab studies (Koren et al. 2014; Raal et al. 2015; Robinson et al. 2014; Sabatine et al. 2015; Stroes et al. 2014) included in-clinic and at-home dosing using the autoinjector. The THOMAS-2 study was the first phase 3 study to use the AMD device, which allows monthly dosing with a single injection. The THOMAS studies found that after proper device administration training, nearly all enrolled patients were able to successfully administer evolocumab in the home-use setting.

The THOMAS studies were open label because blinded allocation to injection devices is not possible. Possible limitations include inaccuracy or bias in self-reporting for the primary endpoint, but the profound LDL-C reduction seen at follow-up in both studies indicates that the proportion of successful self-administrations is reliable.

## Conclusions

The THOMAS-1 and THOMAS-2 studies demonstrate that after appropriate training, patients can safely and effectively self-administer evolocumab in the home-use setting. LDL-C reductions were robust and consistent with expectations from the other PROFICIO studies. AEs, including injection-site reactions, were infrequent and similar between treatment groups. The biweekly and monthly dosing regimens were clinically equivalent.

## Additional file

10.1186/s40064-016-1892-3 Investigator List and Supplemental Data.

## Abbreviations

AE: adverse event
AI: autoinjector
AMD: automated minidoser
CI: confidence interval
CYP3A: cytochrome p450 family 3 subfamily A
HDL-C: high-density lipoprotein cholesterol
LDL-C: low-density lipoprotein cholesterol
NCEP: National Cholesterol Education Program
Q2W: biweekly
QM: monthly
PCSK9: proprotein convertase subtilisin/kexin type 9
PD: pharmacodynamics
PFS: prefilled syringe
PK: pharmacokinetics
PROFICIO: Program to Reduce LDL-C and cardiovascular Outcomes Following Inhibition of PCSK9 In different pOpulations
SD: standard deviation
SE: standard error
T-1: THOMAS-1
T-2: THOMAS-2
