# Supplementary material for: Evolocumab lowers LDL-C safely and effectively when self-administered in the at-home setting
Source: Springerplus. 2016 Mar 9;5:300. doi: 10.1186/s40064-016-1892-3 (PMC4783317; doi:10.1186/s40064-016-1892-3)

Appendix

Study investigators of THOMAS-1 (T-1), THOMAS-2 (T-2), or both (T-1 & T-2)

R. Weiss (T-1 & principal investigator for T-2), C. Recknor (principal investigator for T-2), S. Aronoff (T-1 & T-2), V. Awasty (T-1 & T-2), F. Bacha (T-2), B. Bhagwat (T-1), T. Blevins (T-2), R. Collette (T-2), R. Detweiller (T-1 & T-2), A. Dowell (T-1 & T-2), P. Dzongowki (T-1 & T-2), J. Fialkow (T-1), F. George (T-2), A. Gupta (T-1 & T-2), T. Hack (T-2), E. Hage-Korban (T-1 & T-2), K. Heaton (T-1), S. Kanani (T-1), A. Kivitz (T-1 & T-2), I. Loh (T-1), M. Malone (T-1 & T-2), J. Neutel (T-2), M. O'Mahony (T-2), S. Promisloff (T-1), T. Raoof (T-1), A. Reichman (T-1), T. Repas (T-1), E. Riffer (T-2), H. Roseman (T-2), A. Steinberg (T-1 & T-2), J.C. Stringer (T-1 & T-2), M. Throne (T-1)

Training checklists used by sites in the THOMAS studies

The following steps were given in both studies regardless of the study device:

- Before the training
  - Wait 30 to 45 minutes to allow the prefilled autoinjector(s) to naturally reach room temperature
  - Gather all materials needed for injection (study device, alcohol swabs, cotton ball or gauze pad, adhesive bandage, container, Subject Starter Kit, 2 training units, reset tool)
  - Inspect the study device
- Prepare for injection
  - Explain the purpose of the training (ensure patient is comfortable with self-injection)
  - Review study device instructions for use
- Injection instruction
  - Choose and clean injection site
  - Create a firm surface on injection site
  - Inject evolocumab (see list below)
- After the injection
  - Save used autoinjector and its original packaging or dispose of syringe in sharps container
  - Examine the injection site

The following steps were given for injection for particular study devices:

- Autoinjector
  - When ready, pull the white needle cap straight off
  - Place prefilled autoinjector on injection site
  - Push the prefilled autoinjector firmly on skin at injection site
  - Press the blue start button and release thumb
  - Count slowly to 15 seconds for injection to finish
- Prefilled syringe
  - When ready, pull the gray needle cover straight off
  - Remove the air bubble/air gap
  - Holding the pinched skin, insert the needle into the skin using 45 to 90 degree angle
  - With slow, constant pressure, push plunger all the way down until injection is complete
  - Lift syringe gently from skin
- Automated minidoser (AMD)
  - Clean the bottom of prefilled cartridge
  - Load cleaned cartridge into 3.5 mL AMD
  - Keeping the stretch, place loaded injector onto skin with blue flashing light up, and secure adhesive
  - Press and release the start button
  - Carefully peel used injector off skin and check study drug window

Supplemental Table

Supplemental Table 1. PK/PD analysis of unbound evolocumab serum concentration and PCSK9 levels

**THOMAS-1 (140 mg biweekly)**	**Autoinjector (n = 74)**	**PFS (n = 75)**
Unbound evolocumab serum concentration (PK), mean (SD) µg/mL		
Week 6	4.62 (4.62)	3.12 (3.24)
PCSK9 levels (PD), mean (SD) ng/mL		
Baseline	311.8 (117.5)	312.3 (100.9)
Week 6	145.2 (90.3)	178.9 (100.9)
**THOMAS-2 (420 mg monthly)**	**Autoinjector (n = 82)**	**AMD (n = 82)**
Unbound evolocumab serum concentration (PK), mean (SD) µg/mL		
Week 10	32.9 (21.0)	29.1 (18.1)
Week 12	10.3 (8.8)	7.5 (6.2)
PCSK9 levels (PD), mean (SD) ng/mL		
Baseline	311.2 (96.1)	302.9 (81.3)
Week 10	36.4 (65.4)	33.4 (68.3)
Week 12	226.5 (122.9)	243.3 (114.8)

AMD, automated minidoser; PCSK9, proprotein convertase subtilisin/kexin type 9; PFS, prefilled syringe; pharmacokinetic/pharmacodynamic (PK/PD); SD, standard deviation

Supplemental Figures

Supplemental Figure 1. Diagrams of A) autoinjector, B) prefilled syringe, and C) automated Minidoser

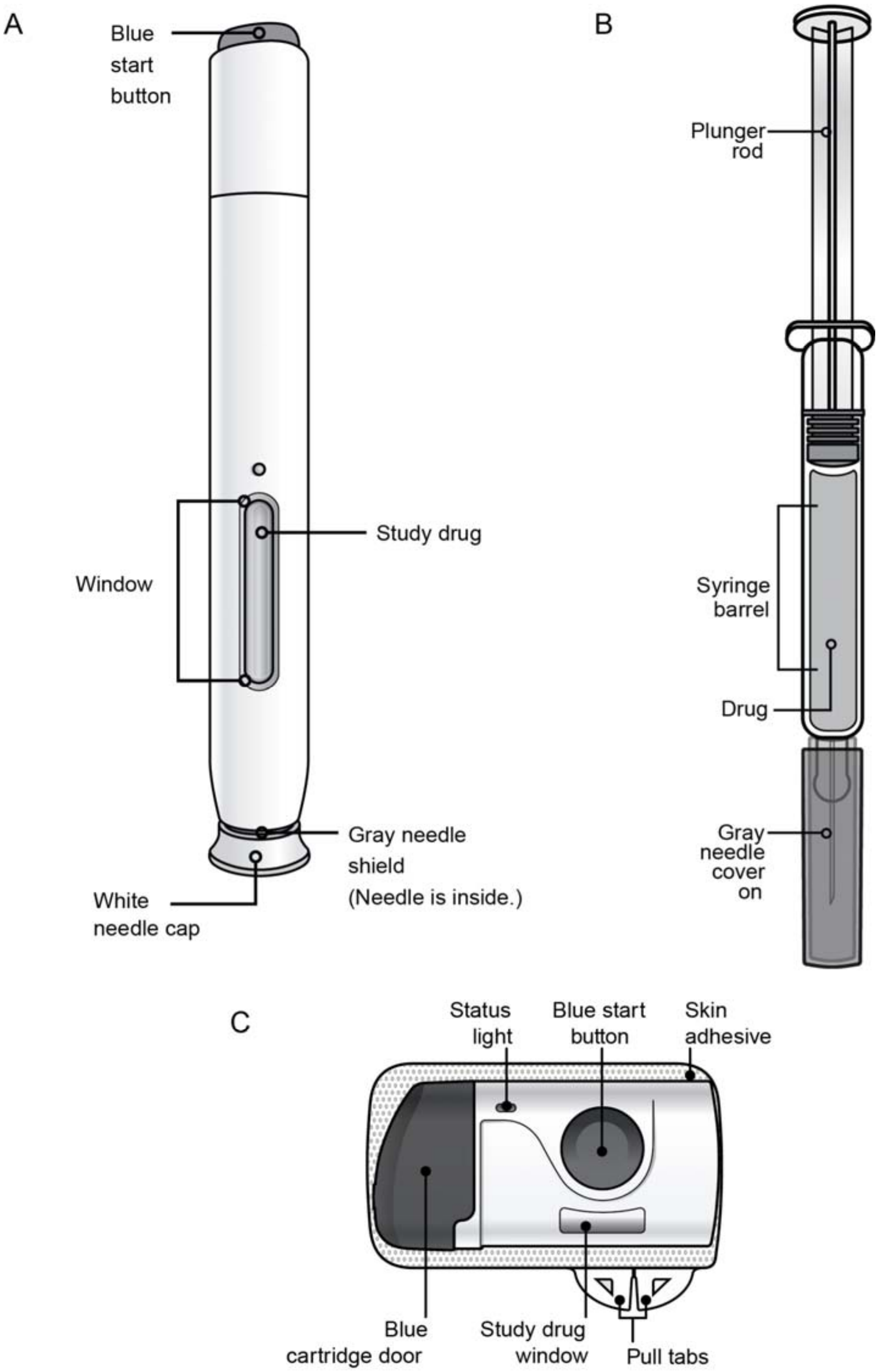

Supplementary Figure 2a. THOMAS-1 Patient disposition. [a]Though the patient completed all screening and efficacy assessments, the patient was prematurely enrolled into the extension study before completing the week 8 follow-up phone call for adverse events.

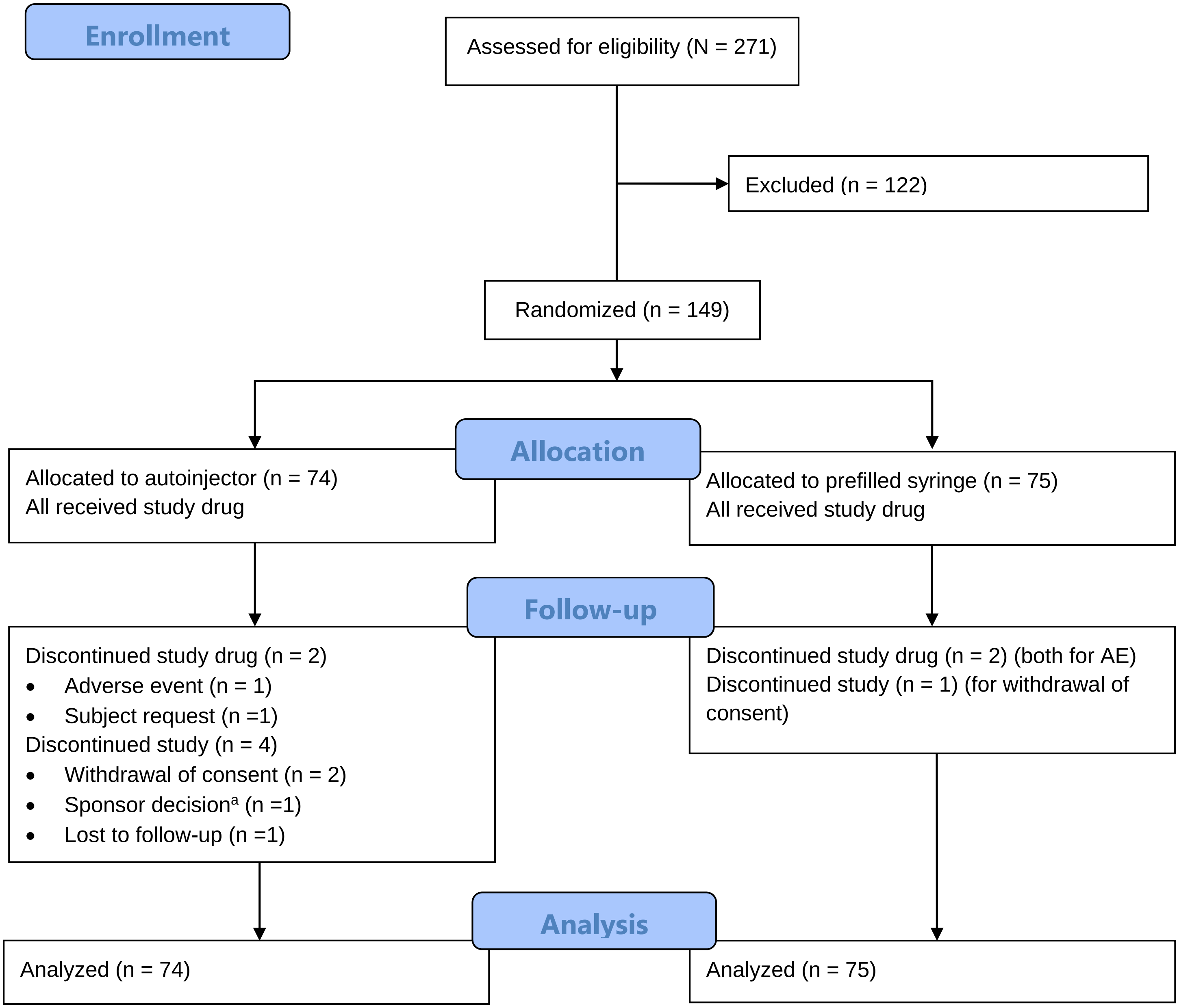

Supplementary Figure 2b. THOMAS-2 patient disposition

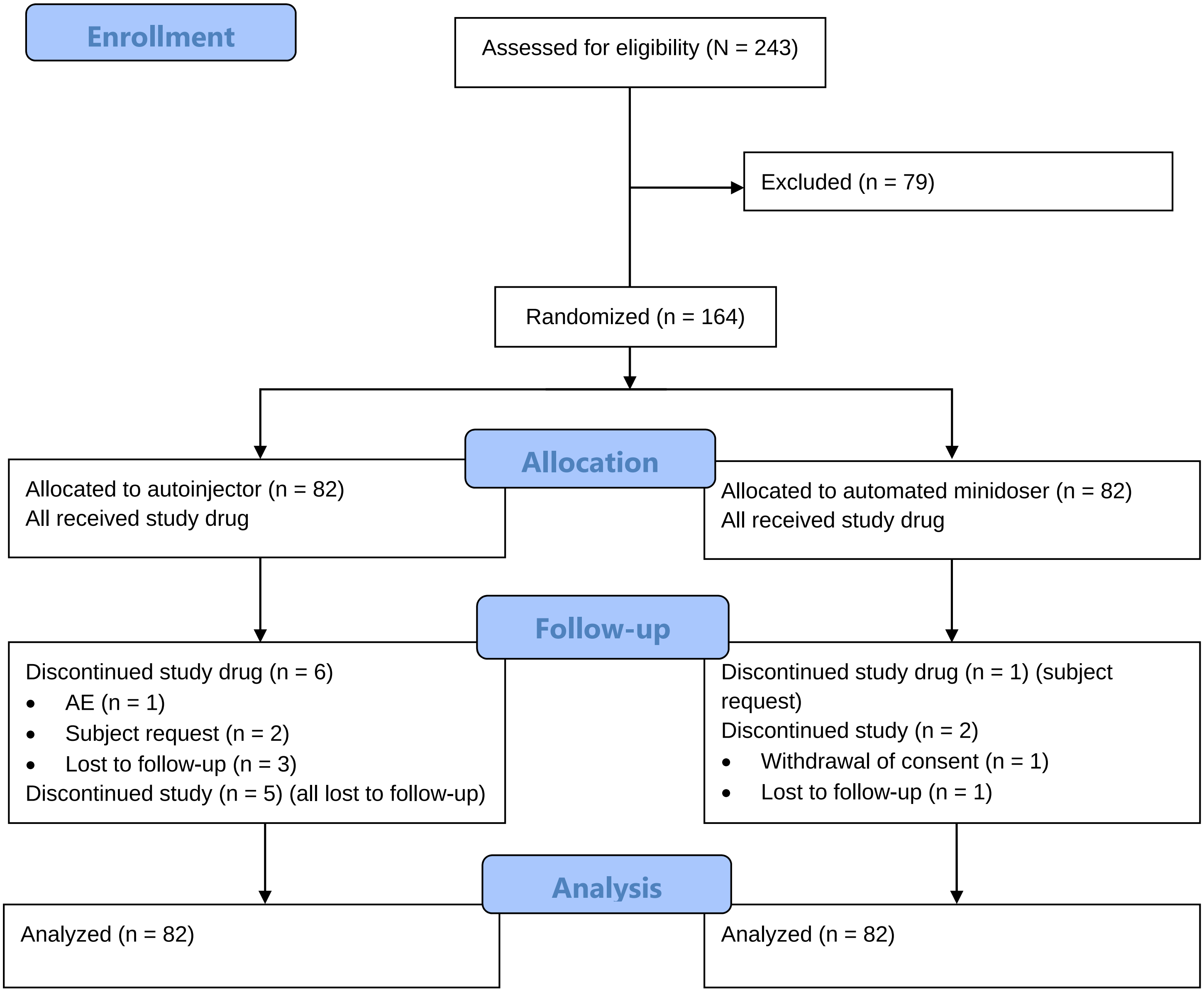

Supplement: Supplementary file 1 — 10.1186/s40064-016-1892-3 Investigator List and Supplemental Data. [file 40064_2016_1892_MOESM1_ESM.pdf]
